# A Pilot Randomized Trial of a Program to Prevent Falls Among Community-Dwelling Adults Living with Mental Illness Demonstrates Feasibility

**DOI:** 10.1177/00469580251327038

**Published:** 2025-04-21

**Authors:** Meryl Lovarini, Lynette Mackenzie, Mandy Meehan, Rebecca Baiada, Nicola Hancock, Justin Scanlan, Megan Swann, Evelyn Argall, Lindy Clemson

**Affiliations:** 1 The University of Sydney, Camperdown, NSW, Australia; 2 Older People’s Mental Health Service (OPMHS), Brookvale, NSW, Australia

**Keywords:** accidental falls, COVID-19, older, aged, mental health

## Abstract

We adapted the Stepping On fall prevention program, conducted a pilot feasibility trial and explored program acceptability. Using a pilot randomized controlled trial (RCT) design, participants from the Older People’s Mental Health Service (OPMHS) Ryde, Sydney were randomly allocated to the adapted program or usual care. Trial participation data, self-reported falls and other fall-related outcomes were recorded. Aspects of program acceptability were recorded in fieldnotes and analyzed using content analysis. Due to the COVID-19 pandemic, recruitment was limited to 1 program only. Eighteen participants were screened, 11 were recruited and randomized (Stepping On n = 6, Usual Care n = 5), 8 returned falls calendars, while 7 provided other fall-related outcome data. Tailoring of the program was valued, however, exercise completion between program sessions was challenging. Evaluation of the adapted program for people living with mental illness using an RCT design demonstrated feasibility, and the program was acceptable to participants. A larger trial is needed to determine program effectiveness.

Trial registration number: ACTRN12619001642178 (Australian New Zealand Clinical Trials Registry).

## Introduction

Falls are common amongst older adults. Internationally, around 35% of adults living in the community aged 65 years are reported to fall annually leading to higher mortality and morbidity.^1^ Fallers may experience physical injuries^2^ as well as a fear of falling, activity avoidance, reduced functional ability, more dependence and lowered quality of life.^3^

Older people diagnosed with mental health conditions have an increased risk of falling.^4^ One study of older British adults living in the community and using mental health services found that they had twice the incidence of falls and the incidence of hip fractures was 4 times higher compared to the general population of older adults living at home.^5^ This is similar for older community mental health users in the United States who report higher rates of falls, and medical emergency visits, as well as longer hospitalizations.^6^ People with mental illness are also exposed to other risk factors for falls such as using analgesics and/or psychotropic medications, cardiovascular disease, hypertension, visual disturbances and syncope.^7,8^ Depression is a common mental health issue and is a well-established risk factor for falls.^9^ Further, anti-depressant medications contribute to fall risk due to their hypotensive and sedative effects.^10^

A higher risk of falls may be present from middle-age for those living with mental illness. For people living with schizophrenia, comorbid physical health conditions and taking prescribed analgesics are linked with more falls and fractures for both middle-aged and older adults.^7^ One review found that falls were experienced by younger mental health inpatients compared to falls reported in medical-surgical units.^11^ This was echoed by a Japanese study that found that 30% of falls in a psychiatric unit involved adults aged under 65 years.^12^ Adults living with mental illness tend to have chronic medical conditions^13^ and have poorer health outcomes and mortality earlier than the general population.^14^ Thus, given the range of risk factors for falling, and at an earlier age, prevention of falls is an important health consideration for this group.

Only 1 study has sought the views of adults aged 50 and over living with mental illness on their experiences of falling.^15^ In this study most participants could not identify the cause of their falls, did not understand of their fall risk and were unlikely to seek help following a fall despite experiencing physical and emotional injury. Many were concerned about falling in the future, but the most common strategy used to prevent future falls was to simply “be careful.”

Clear evidence indicates that falls can be prevented among typical older adults living in the community.^16,17^ However, there is very little evidence of effective community-based fall prevention interventions for adults living with mental illness with limited evidence to guide practice.^18^ As older adults living with mental illness have similar fall risk factors as those who don’t have a mental illness, adapting evidence-based fall prevention programs currently available in the community may offer an appropriate way forward.

Stepping On is a community-based fall prevention program for older adults.^19^ Developed in Australia, this group-based program assists participants in reducing their risk of falling and increasing self-confidence. Incorporating adult learning principles, the program is underpinned by a decision-making framework with a focus on self-efficacy, reflection, problem solving and behavior change. The program content focusses on key risk factors for falls and strategies for addressing those risks. Stepping On is effective in preventing falls, improving self-confidence and increasing the use of protective behaviors,^20^ and has been recommended internationally^21^ and in Australia.^22^ Stepping On has been adapted for stroke survivors,^23^ older adults with mild cognitive impairment^24^ and adults aged over 65 years with a cancer diagnosis.^25^ Improvements in balance and lower limb strength have been reported in middle-aged and older adults participating in Stepping On programs conducted in the clinical mental health setting.^26^ Thus, Stepping On shows promise as a potentially effective intervention for this group. In this study we adapted the Stepping On program tailored to the needs of community-dwelling adults aged 50 and over living with mental illness and evaluated the adapted program in a pilot trial. Our objectives were to investigate the feasibility of the trial methods and explore program acceptability.

## Methods

### Study Design

We conducted a 2-group pilot randomized controlled trial (RCT).^27^ Ethical approval for the study was obtained from North Sydney Local Health District Human Research Ethics Committee, approval number: 2019/ETH10457, and was conducted according to the Helsinki Declaration of 1975 as revised in 2024. The study also met the requirements of the EQUATOR/CONSORT guidelines for reporting a pilot or feasibility trial.^27^ The study was conducted during the COVID-19 pandemic period of 2020 to 2022. All trial procedures and activities complied with relevant mandated COVID-19 government public health orders in place during the trial period. The impact of the COVID-19 pandemic on implementation of the trial is described in the following sections.

### Setting

The trial was conducted in partnership with 1 Older People’s Mental Health Service (OPMHS), in Sydney, Australia. The OPMHS provide a range of clinical services with the aim of improving the mental health, wellbeing and quality of life of older adults living with mental health conditions.^28^

### Participants, Recruitment, and Randomisation

To be included, participants had to have a mental health diagnosis, be receiving services at the Older People’s Mental Health Service (OPMHS), aged 50 years or over, living in the community, be willing to participate in the adapted program, be able to understand and converse in English and provide written informed consent. The criteria of 50 years or over was nominated as patients who fall in mental health units tend to be younger than those not receiving mental health treatment.^11^ Exclusions were experiencing an acute mental health episode, living with a dementia diagnosis or residing in a nursing home.

Potential participants were identified and screened for eligibility by clinical staff at the OPMHS. Those wanting to participate provided written informed consent. Following recruitment, all participants underwent baseline assessment conducted at the OPMHS by 1 member of the research team. Participants were then randomized to either the adapted Stepping On program or usual care group. Using a computer-generated schedule, random allocation was conducted off-site by a different team member using sealed opaque envelopes.

### Development, Design, and Delivery of the Adapted Stepping on Program

The adapted program was developed in consultation with mental health service users at the OPMHS (n = 7) and an expert panel comprising a mental health service user and allied health professionals experienced in fall prevention (n = 5), fall prevention and mental health (n = 2), and mental health (n = 2).

Key features of the adapted program are shown in Table 1 with specific adaptations shown in italics. Balance and strength exercises as recommended by the Otago program,^29^ home hazard risks and solutions as measured by the Home Falls and Accidents Screening Tool—self-report,^30^ community safety,^31^ safe footwear,^32^ medication review,^33^ low vision and falls,^34^ what to do after a fall,^35^ vitamin D and calcium,^36^ and public transport safety.^37^ The adaptations related mainly to program structure, content and delivery. The program was conducted over 8 weekly sessions to provide more time on each topic, permit the inclusion of topics relevant to mental health and ensure sufficient time for exercise practice and progression. The booster session was conducted 1 month after the 8-week program to support program follow-through. A smaller group size was recommended to facilitate participant engagement and learning and enable more focussed attention on individual experiences. Introduced topics focussed on the impact of mental state on falls, social and lifestyle factors, physical health and co-morbidities, medication type and polypharmacy, along with the importance of physical activity. Enhanced topics included medication management, sleep hygiene and overcoming barriers to exercise. All participants received a program bag and program materials. Leg weights were provided where appropriate. Transport to and from the program was provided by staff if needed.

**Table 1.** Outline of the Adapted Stepping On program.^a^.

Conceptual underpinning	Program structure	Key content	Key components
• Adult learning principles	• 8 two-hour weekly group sessions	• Balance and strength exercises	• Program leader facilitates meaningful engagement
			• Peer engagement in solution generation, support and feedback
			• Positive optimism and encouragement
			• Use of story, prompts and cues
			• Exercises and safety strategies linked to fall prevention
			• Self-directed goal-oriented appraisal
			• Exercises reviewed and upgraded
			• Outdoor mobility practice
			• Weekly homework for action planning
			• Review of homework and accomplishments
			• Facilitator/Invited experts’ key messages are relevant to mental health
			• Transport provided for those needing it
			• Stepping On bag and leg weights supplied
			• Modified session agendas, homework, handouts, logbook, displays and props
			• Use of co-facilitators
			• Recognition and acknowledgment of lived experience of mental illness
		• Home hazard solutions	• Increased time reviewing and practising exercises
		• Community safety	
		• Safe footwear	
		• Medication review	
		• Low vision and falls	
		• What to do after a fall	
		• Vitamin D and calcium	
		• Public transport safety	
		• A focus on one major topic per week	
	• < 10 participants per group	• Topics specific to mental health introduced	
	• Booster session at 1 month		
	• Conducted at the OPMHS.		
• Decision-making framework to prompt reflection and action	Participants:		
	• Aged 50 years and over, living with mental illness in the community		
	• Not in an acute phase of mental health condition		
	• Fallen in the past year or who have a fear of falling		
	• Independent walking with or without a walking stick		
	• Cognitively intact		
• Self-efficacy as a tool for change	• Conversational English or language of the group		
• Reflective motivation			
• Problems solving			
• Support follow-through with safety behaviors			

a Adapted from Clemson and Swann.^19^

The program was conducted at the OPMHS by a staff occupational therapist and a visiting physiotherapist, both of whom were experienced in delivering Stepping On programs and working with adults living with mental illness. An additional occupational therapist staff member attended the program providing assistance where required. The program was delivered in accordance with COVID safe protocols. Participants and staff were screened for COVID prior to building entry, the number of people in the program room was restricted due to physical distancing requirements, the program environment and materials were sanitized before and after each session and staff members wore a mask when in close proximity to participants. Participants were provided with their own program space and materials, display items were demonstrated by staff rather than handled by program participants, while morning tea was packaged into single serves and distributed by staff.

### Usual Care Group

Participants allocated to the usual care group continued with their usual medical treatments and health care services with no restrictions placed on their use of health services within or outside of the OPMHS.

### Data Collection

The number of participants screened, recruited and assessed were recorded along with study withdrawals and reasons for withdrawal. Demographic data collection included age, sex, type of residence, living arrangements and the number of health and community services used in the previous 3-month period. Mental health diagnoses, other health conditions and medication data were also collected.

We collected data on self-reported falls, fall hospitalizations and other fall-related outcomes. Self-reported falls were recorded by participants using a 12-month falls calendar provided at the time of the baseline assessment. The 12-month period enabled data collection for 6 weeks before the program, during the 12-week program period and over 34 weeks after program end. Instructions on how to complete and return the calendars were provided by the assessor. Participants were provided with stamped addressed envelopes and asked to return the calendars each month. If the calendar was not returned, the participant was contacted by a OPMHS staff member or a member of the research team and reminded to return the calendar. If preferred by the participant, the calendar was completed with the staff or research team member at the time of the reminder. Fall-related hospital admission data were extracted from each participant’s electronic medical record by the blinded assessor for the 12-month period before the program, during the 12-week program period and at 34 weeks after program end.

Fall risk, self-efficacy, physical performance and fall risk in the home environment were measured using the Falls Risk for Older people in the Community: FROP-COM assessment tool,^38^ the Falls Efficacy Scale-International (FES-I),^39^ the Short Physical Performance Battery (SPPB)^40^ and the Home Falls and Accidents Screening Tool Self Report version (HOME FAST-SR).^30^ The HOME FAST-SR was used as home visits could not be conducted due to COVID-19 related restrictions. Participants were assessed on all 4 outcome measures at the OPMHS by the blinded assessor before and after the 12-week program period.

To explore program acceptability, we recorded the number of Stepping On program sessions conducted along with participant attendance and reasons for non-attendance. The program facilitators recorded in fieldnotes what aspects of each session worked or did not work well as well as participant and facilitator reflections at program end.

### Data Analysis

All quantitative outcome data were analyzed descriptively using SPSS Statistics (version 26). The Mann Whitney non-parametric test for independent samples was used to analyze differences between groups at 12 weeks on the FROP-COM, FES-I, SPPB, and HOME FAST-SR with a significance level set at .05. Outcome data at 12 and 34 weeks were analyzed according to group allocation using complete case analysis.^41^ For program acceptability, we counted the number of program sessions conducted and calculated the rate of session attendance by dividing the number of participants attending by the intended number of participants. A content analysis^42^ of the fieldnote data was conducted using NVivo (version 12) by 2 members of the research team.

### Sample Size

Our initial aim was to conduct 4 programs with an intended total sample size of 40. This sampling strategy comprised 10 participants at 4 OPMHS sites, with 5 participants each randomly allocated to the adapted program or usual care groups. One site could not conduct a program due to staffing and resource constraints, while another could not provide participant transport. Given time limitations to conduct our study and the ongoing impact of COVID-19, we were unable to seek additional study sites. Thus, our trial was then limited to 2 sites, with an intended sample size of 20 participants. After further extensive time delays caused by the COVID-19 pandemic, the first program commenced at 1 site in 2021. The program could not proceed at the second site due to COVID-19 related restrictions on the resumption of in-person group-based programs. Due to these circumstances, participant recruitment was limited to 1 program at 1 site only.

## Results

### Participant Recruitment and Characteristics

**Figure 1.**
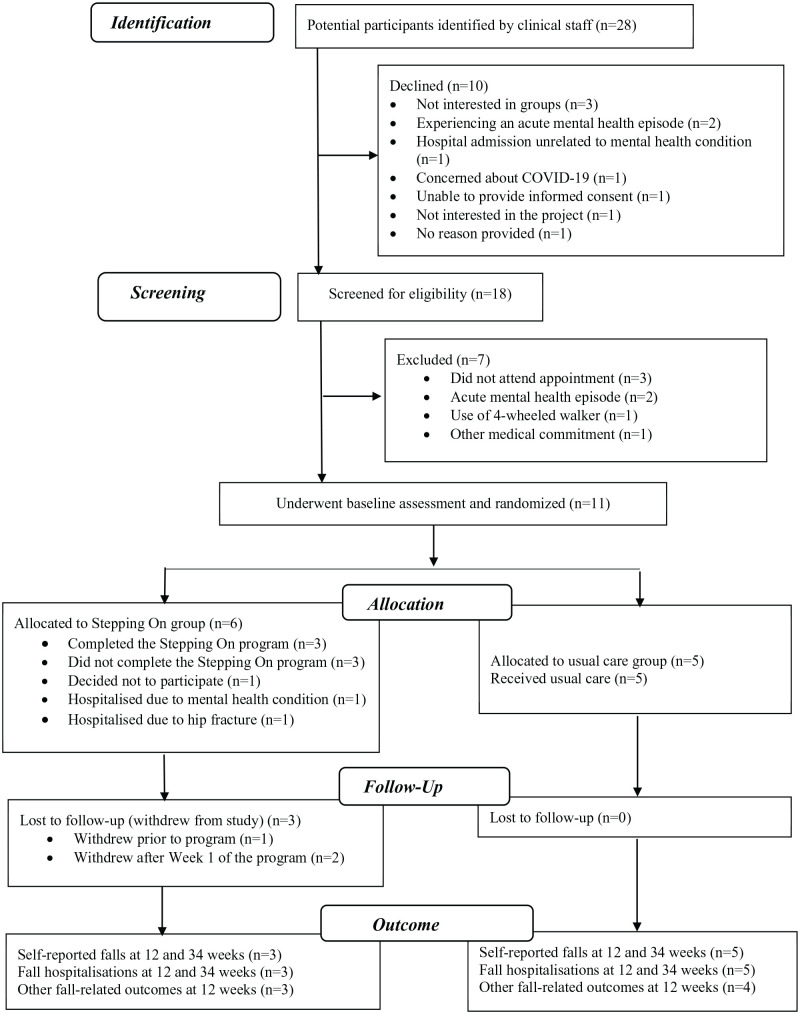
Participant recruitment and study flow.

Participant recruitment and study flow are presented in Figure 1. Twenty-eight potential participants were approached, with 10 declining. Following screening, 11 participants were recruited, underwent baseline assessment and randomized to either the adapted Stepping On program (n = 6) or usual care (n = 5).

Three participants out withdrew from the Stepping On program (intervention group) leaving 3 in the study. One participant withdrew before the program commenced. Two participants were hospitalized after Week 1 of the program. One due to their mental health condition, the other due to a hip fracture following a fall occurring at the participant’s home. Three participants completed the program and there were no study withdrawals from the usual care group.

Participant characteristics are shown in Table 2. Participants were aged between 62 and 76 years with most living alone in an apartment. Participants had an average of 7 health conditions, the most common mental health condition was bipolar affective disorder, and most participants took antipsychotic medication. Eight participants reported 1 or more falls in the previous 12-month period before the program with no difference between groups.

**Table 2.** Participant Characteristics at Baseline (n = 11).

Characteristic	Stepping On (n = 6)	Usual care (n = 5)
Age (mean, range)	69.33 (62-75)	72.20 (68-76)
Female	4 (66.67%)	2 (40%)
Male	2 (33.33%)	3 (60%)
Residence type		
House	2 (33.33%)	1 (20%)
Apartment	4 (66.67%)	3 (60%)
Hostel	0 (0%)	1 (20%)
Living arrangements		
Lives on own	4 (66.67%)	5 (100%)
With partner	0 (0%)	0 (0%)
With family	2 (33.33%)	0 (0%)
Primary mental health condition		
Bipolar affective disorder	3 (50%)	3 (60%)
Depression	1 (16.67%)	1 (20%)
Schizoaffective disorder	1 (16.67%)	1 (20%)
Schizophrenia	1 (16.67%)	0 (0%)
Total no. of health conditions (mean)	6.50 (3-11)	8.20 (5-14)
Medications		
Antipsychotic medication use	4 (66.67%)	5 (100%)
Antidepressant medication use	3 (50%)	3 (60%)
Marital status		
Single	3 (50%)	3 (60%)
Married (defacto)	1 (16.67%)	0 (0%)
Widowed	1 (16.67%)	1 (20%)
Divorced (separated)	1 (16.67%)	1 (20%)
Service use		
Number of health/community services used	3.50 (2-5)	5.80 (1-9)
Receives government funded home support	1 (16.67%)	2 (40%)
Fall-related characteristics		
Self-reported falls in previous 12 months	4 (66.67%)	4 (80%)
Fall-related hospital admissions in previous 12 months	1 (16.67%)	0 (0%)
Falls risk (FROP-COM)	15.60	17.40
Falls self-efficacy (FES-I)	24.67	22.60
Physical performance (SPPB)	8.60	6.60
Falls risk in the home environment (HomeFast)	8.83	10.20

### Electronic Medical Record Reviews

Diagnostic, medication and fall-related hospitalization data were extracted from the electronic medical records for 8 (73%) participants at 12 and 34 weeks. The purpose of this was to validate any self-reported data or to collect data that was not known. The electronic records comprised 2 different platforms with multiple areas within each platform needing to be searched. The average time needed to extract all data was 2.5 h per participant. Group allocation was revealed for 1 participant during the review process however all other outcome data had been collected for that participant in a blinded manner prior to the time of the electronic record review.

### Self-Reported Falls

The falls calendars were returned by 10 (91%) participants during the 6-week pre-program period and 8 (73%) during the 12-week program and 34-week post-program periods. Of the 98 calendars returned over the entire 12-month period, 57 (58.16%) were returned without a reminder, while 41 (41.84%) were returned after reminding or completed with staff from the OPMHS or the research team at the time of the reminder. There were slightly fewer falls in the adapted program group compared to the usual care group at 12 and 34 weeks (Table 3).

**Table 3.** Number and Characteristics of Self-reported Falls.

	6-Week pre-program period participants (n = 11)		12-Week program period participants (n = 8)		34-Week post-program period participants (n = 8)		
Characteristic	Stepping On n = 6	Usual care n = 5	Stepping On n = 3	Usual care n = 5	Stepping On n = 3	Usual care n = 5	Total (%)
Number of falls	1	2	3	4	5	6	21 (100)
Fall location							
Indoors	1	1	2	2	4	6	16 (76.2)
Outdoors	0	1	1	2	1	0	5 (23.8)
Fall type							
Fall getting in/out of bed	0	1	0	1	2	4	8 (38.1)
Trip over carpet/floor item	0	0	0	1	2	1	4 (19.0)
Other^a^	0	1	3	3	1	0	8 (38.1)
Unknown	1	0	0	0	0	0	1 (4.8)
Fall-related injury							
No injury	1	2	1	1	2	3	10 (47.6)
Bruising	0	0	1	3	0	0	4 (19.0)
Knee & upper limb injury	0	0	1	0	3	0	4 (19.0)
Other/not reported	0	0	0	0	0	3	3 (14.4)
Actions taken following fall							
Not reported	1	2	2	3	4	0	12 (57.1)
Moved item from floor	0	0	0	1	0	1	2 (9.5)
Will speak to local doctor	0	0	0	0	0	2	2 (9.5)
Being more careful	0	0	1	0	0	1	2 (9.5)
Other^b^	0	0	0	0	1	2	3 (14.4)

a Other fall types included trip over furniture (n = 1), slip on wet floor (n = 1), getting out of car (n = 1), getting off bus (n = 1), legs giving way (n = 1), vision blocked when walking outside (n = 1), walking on uneven surface (n = 1), running to answer the phone (n = 1).

b Other actions included pausing before standing from bed (n = 1), moving hostel room for 1 day (n = 1), using handrails (n = 1).

Most falls occurred indoors (n = 16, 76.2%). A third of all falls involved getting in or out of bed (n = 8, 38%), followed by trips on carpet or an item on the floor (n = 4, 19%). Eleven falls (52.4%) resulted in injury. The most common injuries were bruising (n = 4, 19%) and injury to the knee and upper limb (n = 4, 19%). Actions taken following a fall were not reported for approximately 60% of all the reported falls, while the most common actions taken following the fall were to move items (n = 2, 9.5%), be more careful (n = 2, 9.5%) or speak to their local doctor (n = 2, 9.5%; Table 3).

### Fall-Related Hospital Admissions

Data on fall-related hospital admissions were available for 11 (100%) participants for the 12-month period before the program, for 10 (91%) participants during 12-week program period and for 8 (73%) participants during the 34-week period after the program. Two fall-related admissions were identified from the electronic medical record review. One admission occurred during the 12-month period before the program for 1 participant from the adapted program group. The other occurred during the program period for 1 participant also allocated to the adapted program group. The admission occurred during Week 2 of the program, following a fall at home resulting in a hip fracture.

### Other Fall-Related Outcomes

Data collection for all other fall-related outcomes took approximately 60 min per participant. Outcomes at program end are presented in Table 4. Outcome data were available for 7 participants (87.5%), with data from 1 participant allocated to the usual care group not collected due to a hospitalization for mental health reasons. There were no differences in outcomes between the 2 groups at the end of the program.

**Table 4.** Other Fall-related Outcomes at 12-Weeks (Program End).

Measure	Stepping On group (n = 3)		Usual care group (n = 4)		*P*-value
	Mean	95% CI	Mean	95% CI	
FROP-COM	15.33	[10.16, 20.50]	13.50	[8.91, 18.09]	.629
FES-I	21.67	[3.70, 39.64]	20.75	[16.57, 24.93]	.857
SPPB	9.00	[2.43, 15.57]	7.75	[5.36, 10.14]	.629
HOME FAST-SR	8.67	[7.23, 10.10]	10.00	[8.70, 11.30]	.114

### Program Acceptability

All program sessions were conducted. Weekly program attendance ranged from 33% to 100%, with 100% attendance at 6 of the 9 program sessions. Reasons for not attending a session included attendance at medical appointments that could not be rescheduled or the impact of the participants’ mental state on the day of the session.

Aspects of the adapted program that worked well included the enhanced emphasis on ensuring participant comfort in group participation and discussions, the greater use of physical props and visual materials, ensuring that each participant had their own program space and materials, having a standard session format that was repeated each session and having the additional program session. Onsite liaison between the program facilitators and relevant OPMHS staff before and after each program session was useful in tailoring each session and encouraging participant follow-through.

Program challenges included some technical difficulties with the microphone and projector, needing more time to ensure correct exercise technique and a lack of suitable handouts on chronic disease. Participants were also unable to physically share program materials or try equipment firsthand during the sessions due to the prevailing COVID-19 restrictions. Challenges in completing the exercises included the presence of knee and back pain, remembering to do the exercises, not using correct techniques and a lack of motivation. While the provision of the adapted program logbook was viewed positively by participants, completion of the logbook, and homework tasks between program sessions was sporadic and inconsistent.

At program end participants reported a range of actions taken as a result of participating in the program including home visits by an occupational therapist (n = 2), referral to an exercise physiologist (n = 2), changed exercise set-up including reminders (n = 2), visit with an exercise physiologist and dietician (n = 1), and receiving a new chair to help with mobility (n = 1). Staying positive, practising in a group environment and having ongoing support from their case manager at the OPMHS were all considered important. Having a more individualized program tailored to their needs was also valued.

For the program facilitators, being mindful of the participant’s life experiences and providing sufficient time and flexibility for participants to share their experiences were important. Recommendations for enhancing program acceptability included conducting a more comprehensive assessment of participants prior to the program to enable for example, early identification of barriers to exercising and the use of simple messaging strategies to reinforce key session content and promote homework completion. Developing more formalized support systems for participants (eg, working more closely with case managers) during and after the program to promote follow-through with gains made during the program was also recommended.

## Discussion

This study is the first pilot randomized trial investigating the prevention of falls among older adults living in the community with mental illness. Although severely disrupted by the COVID-19 pandemic, our trial has resulted in some important insights.

We were able to successfully recruit and randomize participants. A willingness to be part of this trial shows the importance of fall prevention for our participants and supports the inclusion of older adults living with mental illness in future larger fall prevention trials. Forty-two percent of participants needed a reminder to complete the falls calendars which is higher than the 30% reminder rates reported in fall prevention studies involving the general population of older adults.^43^ Actions taken following a fall was missing on nearly two-thirds of the calendars returned by both groups. To enable a more complete dataset in future studies, additional resources will be needed for more frequent calendar reminders and assistance with calendar completion.

Data extraction from the electronic medical records was time intensive. The required information was fragmented across different sections of the records, making the data extraction process onerous. Despite these challenges, medical record reviews are important for confirming diagnostic, medication, and hospitalization data. Given that electronic medical record systems may differ at different sites we recommend that researchers trial the data extraction process to more accurately determine the time and resources needed to complete this task. Although our study was not designed to demonstrate differences in outcomes at the end of the trial, the outcome measures conducted onsite took a short time to administer and were acceptable to participants as indicated by attendance at the follow-up assessments.

We were able to adapt the Stepping On program and deliver the program on site in accordance with COVID-19 safe protocols in place at that time. All program sessions were delivered, and attendance rates were similar to established Stepping On program benchmarks. At program end, our participants reported a range of actions taken as a result of their program participation. The uptake of fall prevention behaviors following Stepping On participation has also been reported by older adults not living with mental illness.^44^ Sustaining new behaviors over time however is challenging, requiring context-dependent support.^45^ Given the range of health professionals that may be involved with older adults living with mental illness, the use of care plans following Stepping On programs may offer a useful way of providing ongoing and specific support for achieving program follow-through. If used, care plans should be developed with participants, distributed with participant permission and adhere to established models of mental health service delivery. Including participant interviews in future studies should be considered, to more fully explore preferred options for support post-program.

Due to restrictions associated with the COVID-19 pandemic, we conducted the program at 1 site only. While not possible in this study, remote delivery of community-based fall prevention programs during the pandemic period have been reported^46^ and online mental health service delivery has increased.^47^ While online program delivery aimed at older adults is promising,^48^ tailored support to enhance digital participation is needed. Our focus in this study was on adaptation and preliminary evaluation of the Stepping On program. Future studies will need to consider program fidelity. Dissemination and scale-up studies of Stepping On have shown that program effectiveness can be maintained providing there is fidelity to key program elements. To assist program facilitators, a range of fidelity tools are available at https://www.steppingon.com/fidelity-coaching-tool-for-stepping-on.

Limitations of this study included the small sample size and the relatively high dropout rates of 50% for the intervention group. Dropouts could be related to the COVID risks or a lack of motivation amongst the participant group. These would impact any generalization of the findings but may be a typical issue for future researchers to plan for when providing a program for people living with a mental illness. As the sample size was small significant quantitative findings about the fall outcomes between groups could not be determined. However, the qualitative findings were positive. Future studies would need to develop targeted recruitment strategies, ways of supporting adherence for study participants to prevent dropouts where possible, and comparison of intervention and control groups at baseline and follow up to exclude any systematic differences.

## Conclusion

The Stepping On program can be adapted to meet the needs of older adults living with mental illness in the community. The program was acceptable to participants and evaluation of the program using an RCT design was feasible. A larger trial is needed to determine program effectiveness. Whilst there were limitations, the study has provided a road map for designing a larger RCT study for researchers.
